# Bioeconomy from experts’ perspectives – Results of a global expert survey

**DOI:** 10.1371/journal.pone.0215917

**Published:** 2019-05-01

**Authors:** Irwa Issa, Sebastian Delbrück, Ulrich Hamm

**Affiliations:** 1 Department of Agricultural and Food Marketing, Faculty of Organic Agricultural Sciences, University of Kassel, Witzenhausen, Germany; 2 BIOCOM AG, Berlin, Germany; Central Agricultural University, INDIA

## Abstract

Effective global collaboration is crucial to achieving the UN Sustainable Development Goals (SDGs). It requires an understanding of the needs of individual countries and their expectations related to bioeconomy. With the aim to explore the prospective developments in the global bioeconomy over the next 20 years, the German Bioeconomy Council, an independent advisory body to the German Federal Government, commissioned BIOCOM-AG to invite experts from around the globe to share their insights in a global expert survey. The survey was conducted online in autumn 2017. 345 experts from 46 countries completed the questionnaire about future developments and strategies in the global bioeconomy. As claimed by the experts, the upcoming bioeconomy must primarily meet humanity’s needs in the energy, agriculture, and food sectors. Moreover, innovative products based on renewable resources are anticipated to be of great importance. Even though all UN SDGs will be affected by future bioeconomy success stories, five SDGs stood out within the sample: SDG 12: ‘responsible consumption and production’; SDG 9: ‘industry, innovation and infrastructure’; SDG 13: ‘climate action’; SDG 7: ‘affordable and clean energy’; and SDG 11: ‘sustainable cities and communities’. About three quarters of the experts emphasized the need to specifically address three conflicting goals in any future bioeconomy strategy: non-food uses of arable land, use of crop land to produce feedstock for meat, milk and egg production and, finally, the conversion of virgin forests into agricultural land. Most experts stated that reducing food loss and waste is crucial to eradicating the world hunger problem. The proposed solutions relied greatly on innovation and technological development. Bioeconomy expertise and know-how should be shared in close cooperation between developed and developing economies to reach UN SDGs. A supportive political framework would be the ultimate goal towards furthering the progress of a future bioeconomy all over the world.

## 1 Introduction

Bioeconomy refers to the subdivision of the economy, based on biology and biosciences [1,2]. Yet, there is no universally accepted definition of bioeconomy. The German Bioeconomy Council (GBC), an independent advisory body to the German federal government about topics related to the future bioeconomy, defined bioeconomy as *“the production and utilization of biological resources (including knowledge) to provide products*, *processes*, *and services in all sectors of trade and industry within the framework of a sustainable economy”* [3]. Thus, bioeconomy is an approach aiming to substitute conventional synthetic functions (e.g. fossil-resources-based products) by applying new innovative technologies which rely primarily on biological processes such as fermentation, make use of advances in biotechnology and molecular biology, and build upon terrestrial or aquatic biomass [4,5]. Bio-based products originate from a wide spectrum of industries including agriculture, forestry, fishery, feed and food, paper and pulp, building and construction, together with the biotechnology, and bio-based energy, chemical, and pharmaceutical industries [4,6–9].

Global trade in bio-based products has been booming over the last decade. While bio-based products represented about 10% of total international trade in 2007, this figure reached 13% in 2014 [2]. The data available indicate that the turnover of bio-based products surpassed US$2,500 billion worldwide in 2014, of which the European Union has the largest share (about US$2,200 billion) while the United States’ bio-based industries (excluding food) represent around US$370 billion. Equivalent data about bio-based products from other nations are very scarce [2,9,10]. Nevertheless, an increasing number of countries (currently about 50) are proposing to reinforce and strengthen the position of bio-based products in their economies [11].

Bioeconomy and sustainable development became two central pillars of many countries’ national policies [10,12]. There are, however, compound tensions (sometimes even contradictions) between these two pillars since the current concept of bioeconomy does not necessarily meet the criteria of the Sustainable Development Goals (SDGs) endorsed under the UN conventions on biodiversity and CO_2_ neutrality by 2050 in the context of the Paris climate agreement [13]. From the current bioeconomy perspective, most economic analyses and policy-driven strategies primarily consider the profitability and market potential along with (selective, but not all) societal aspects. Though current bioeconomy basically functions and operates on the utilization of available natural resources, the environmental and ecological costs have been taken into consideration much less and the depletion of natural resources has often been overlooked [5], such as the intensive cultivation of oil palms to produce biofuel and the enormous environmental repercussions on tropical rainforests and biodiversity loss that this has had (e.g. [14–17]). Similarly, land use changes and increasing cultivation of biofuel crops (such as maize and sugarcane which are food crops) pose risks to ecosystems and biodiversity, as well as fluctuations in food prices with the ensuing negative impacts on social welfare (e.g. [18–21]).

A crucial issue is, therefore, to question and examine the sustainability of bioeconomic innovations and to determine whether they do undoubtedly embody a footstep in the right direction towards achieving a circular bioeconomy. Whether the cycles come full round to reach the all-encompassing goal of an ecological “zero waste” and sustainable ecocycles of “zero emission” [10,22,23]. UN SDGs should therefore be considered a priority and the basis for any evolving bioeconomic innovations [5,24,25]. In literature, a few principles such as ecological design as a large concept [26], principles of ecological economics (e.g. [27,28]), biomimicry thinking, methodology, and tools for adapting biomimicry innovations inspired by nature [29], and designing regenerative cultures [30], among others, have been explicitly or implicitly highlighted for a real transition towards sustainable bioeconomy. The abandonment of the “unlimited economic growth” and “business-as-usual” concepts should also be replaced with a resilient system that is ecologically sustainable and socially just [1,2,30,31], and includes more reliance on bio-based mimicries that back and maintain ecological innovations rather than abusive approaches of technology [5,30,32,33].

The common global policy agenda is to reach sustainable bioeconomy which supports the UN SDGs. This can be boosted mainly through further improvements in the global bioeconomy that are directed at sustainability, inclusive transformation, and well-being. Considering the different conditions under which national economies operate, the way forward and the contribution of bioeconomy can differ significantly according to the countries under examination. Industrial countries in Europe and North America perceive bioeconomy as a competitive, resource-efficient, low-carbon economy, as well as an opportunity to develop innovative bio-based products and processes. It is seen as a tool for creating jobs and opening up new markets. Meanwhile, emerging economies in Asia, Africa, and Latin America are making use of their huge biological resources, while at the same time investing in the development of new industrial segments. Many developing countries, on the other hand, are seeking to reinforce and foster sustainable livelihoods through bio-based networks and to build partnerships for technology transfer [2,34]. Irrespective of countries and development status, the shift toward bioeconomy will primary depend upon the (bio)technological advancement of an array of processes and bio-based innovations. It will count on reaching a breakthrough in terms of technical performances and cost effectiveness, and ultimately rely on the availability of sustainable biomass [10].

Effective global collaboration is crucial to achieving the UN-endorsed SDGs [35,36]. It requires an understanding of the needs of particular countries alongside with their expectations related to bioeconomy. Expert insights into the driving forces and existing obstacles associated with bioeconomy provide a valuable base point for global policy makers. Against this background, intuition into outlooks, drivers, and barriers associated with sustainable bioeconomy in different countries around the globe are indispensable for further international strategic directions, policy options, and collaboration in this domain. The main objective of the present study is to provide insights into future opportunities and developments in the bioeconomy around the world from experts’ perspectives.

The article is structured as follows: first, design and implementation of the expert survey are illustrated, second, the major results followed by a brief discussion, are given. The last section provides conclusions and recommendations for the future of global sustainable bioeconomy.

## 2 Material and methods

### 2.1 Design and implementation of the expert survey

With the aim to explore the prospective developments in the bioeconomy from an expert point of view, the GBC commissioned BIOCOM AG to invite experts from around the globe to share their insights within the framework of a global expert survey. The questionnaire for this purpose was developed by the members of GBC during their regular meetings. Experts from 46 countries with established bioeconomy or bioeconomy-related schemes [13] were invited to take part in this survey. The global expert survey also incorporated participants representing EU institutions and international organisations (such as the UN and FAO). To represent the bioeconomy in general, the backgrounds of the experts and officials invited vary considerably, representing diverse sectors (e.g. agriculture, forestry, food, biotechnology, health) including participants with different roles (e.g. researchers at public institutions, members of national bioeconomy advisory councils, policy makers, and representatives from industries, NGOs, associations, and other civil society organizations).

Designed and administered using the open source online tool LimeSurvey, the survey allowed the large number of global participants to fill in the questionnaire at their own convenience. The respondents were invited to share their thoughts on a number of topics related to future developments in the bioeconomy. To this end, the questionnaire combined closed-ended and open-ended questions, with all relevant questions being compulsory. Answer categories were randomized to avoid a sequence effect. After a pre-test with 14 experts, the questionnaire was improved. In autumn 2017, an email invitation with a personalized link to start the online survey was sent to the pre-identified experts. A total of 4,331 experts were invited to participate and 345 experts completed the questionnaire in full. The response rate of 8% is a relatively high ratio for such a survey method [37].

### 2.2 Data analysis and clustering procedure

Data analysis was conducted in two ways, globally and clustered by country. Clustering was performed with an aim to highlight specific group traits which could not be recognized in the global sample. Content analysis [38–40] was applied to qualitative data in two steps. First, relevant categories were built by team members in Microsoft Excel. Second, the coded data were tabulated based on word frequency to identify the main categories and according to clusters of countries.

For the purpose of comparison, we classified the countries in our international sample according to the World Bank Atlas method [41]. The World Bank categorizes the member countries (189) and all other economies with populations of more than 30,000. For operational and analytical purposes, countries and territories are principally divided into “Income Groups” according to 2016 Gross National Income (GNI) per capita, calculated using the World Bank Atlas method. The groups are ‘Low-Income, $1,005 or less’; ‘Lower-Middle-Income, $1,006–3,955’; ‘Upper-Middle-Income, $3,956–12,235’; and ‘High-Income, $12,236 or more’ [41]. According to the latest metadata of World Bank in 2018 [42], 52 countries and territories are classified under ‘High-Income Group’ (GNI per capita according to the Atlas method is $12,236 or more). However, in our sample, 23 out of 46 countries (or 201 out of 315 interviewed experts, when ignoring the 30 experts who work at European or International Organisations), fall within this group. By using the median, we split this group into two further sub-groups: ‘High-Income Economies, $12,236–32,290’ and ‘Very-High-Income Economies, $32,291 and more’. The idea behind this procedure was to make the generated clusters more homogenous (within clusters) and more heterogeneous (between clusters). Similarly, we merged the Low-Income countries and the Lower-Middle-Income countries into one cluster ‘Low- and Lower-Middle-Income Economies, up to $3,955’, since only a few observations from each were represented in our sample. Moreover, the interviewed experts who work for European and international organisations were also amalgamated into one cluster. The resulting 5 clusters are presented in Table 1.

**Table 1:** Clustering respondents according to the Gross National Income (GNI) of their countries. All respondents n = 345 (Adopted from World Bank GNI Atlas Method* [41]).

Cluster	Description	Countries (Number of respondents)
1	**Low- and Lower-Middle-Income Economies (up to $3,955 per capita), (n = 36)**	India (5), Indonesia (6), Kenya (8), Mozambique (1), Nigeria (6), Sri Lanka (4), Tanzania (2), Uganda (4)
2	**Upper-Middle-Income Economies ($3,956–12,235 per capita), (n = 78)**	Argentina (9), Brazil (18), China (1), Colombia (8), Malaysia (3), Mauritius (2), Mexico (6), Namibia (7), Paraguay (3), Russia (2), South Africa (7), Thailand (12)
3	**High-Income Economies ($12,236–32,290 per capita), (n = 42)**	Italy (13), Latvia (6), Lithuania (4), Portugal (1), South Korea (3), Spain (12), Uruguay (3)
4	**Very-High-Income Economies ($32,291 and more per capita), (n = 159)**	Australia (16), Austria (11), Belgium (5), Canada (5), Denmark (18), Finland (15), France (5), Germany (26), Iceland (2), Ireland (6), Japan (6), Netherlands (14), New Zealand (2), Norway (10), Sweden (14), UK (8), USA (9)
5	**European and International Organisations (n = 30)**	Europe (16), International (14)

* **World Bank Atlas method:** GNI per capita is the gross national income, converted to U.S. dollars using the World Bank Atlas method, divided by the midyear population. GNI is the sum of value added by all resident producers plus any product taxes (less subsidies) not included in the valuation of output plus net receipts of primary income (compensation of employees and property income) from abroad. GNI is calculated in national currency but is usually converted to U.S. dollars at official exchange rates for comparisons across economies [41].

## 3 Results

### 3.1 Descriptive statistics and sample description

345 of the invited experts completed the questionnaire. The respondents represent 44 countries in addition to those working for European institutions and international organisations, while no responses came from Mali and Senegal. Table 2 shows that almost all respondents were engaged in research, development, business, or policymaking related to the bioeconomy. It is worth noting that 28% were members of a national bioeconomy advisory council. The expert’s main areas of operation cover a variety of sectors mentioned in the definition of bioeconomy provided by the European Commission [6], such as agriculture (22%), biotechnology (17%), and energy (10%), including a number of additional sectors as well as representatives from EU institutions and international organisations (UN, FAO). Table 2 also indicates that 44% of the respondents described their roles as researchers at a public institution and 24% defined themselves as policy makers, public officials, or public administration staff. A further 20% of experts belonged to the industrial sector (including owners or managers of private companies), 8% were representatives of a civil society organization including NGOs, and another 2% represent experts belonging to an association.

**Table 2:** Descriptive statistics of respondents (N = 345).

Descriptive statistics	Categories	Percent (N = 345)
**Engagement in research, development, business or policymaking related to the bioeconomy**	Yes	95,4
	No	4,6
**Membership of a formal/governmental bioeconomy advisory council?**	Yes	28,1
	No	71,9
**Expert’s main role**	Researcher/ lecturer at a public institution (university, research institute)	43,8
	Policy maker/ public official/ public administration staff	24,3
	Industry	19,7
	Representative of a civil society organization/ NGO	7,8
	Association	2,0
	Other	2,3
**Expert’s main sector of operation**	Food, nutrition	4,1
	Fishery	3,5
	Biotechnology	17,4
	Health, pharma	1,2
	Energy	9,9
	Agriculture	22,3
	Chemistry	6,4
	Wood and paper manufacturing	3,5
	Forestry	7,2
	Other	24,6

### 3.2 Upcoming bioeconomy success stories

**Fig 1:**
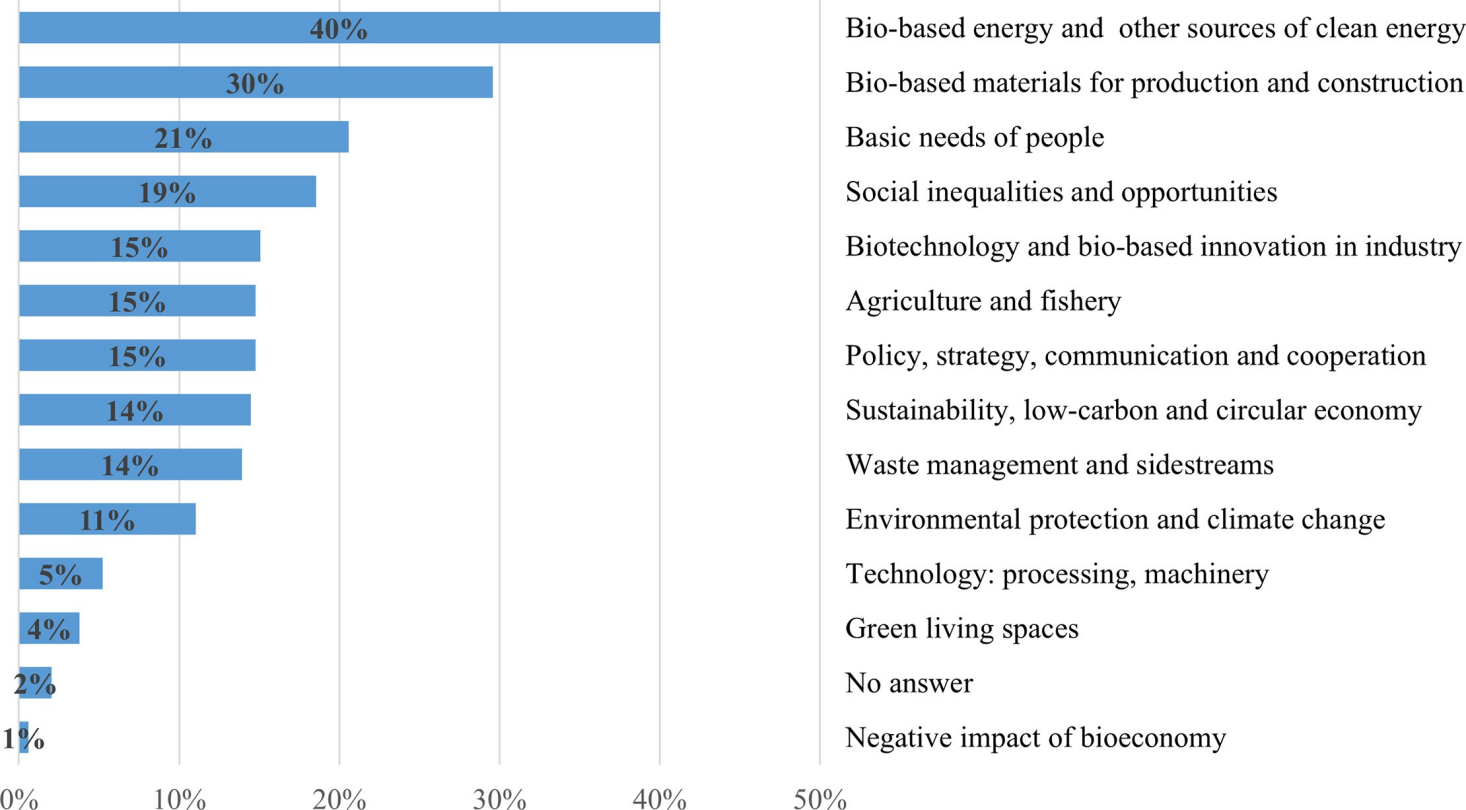
Main promising success stories of the bioeconomy over the next 20 years (n = 345). Note: multiple answers.

The experts were asked to name the three most promising bioeconomy success stories for their respective countries over the next 20 years and the corresponding UN SDGs that will be affected. 98% of the respondents mentioned one promising success story, while 78% and 64% of them additionally stated second and third promising success stories, respectively. As per the 827 expert opinions collected, the upcoming bioeconomy will meet humanity’s needs in different domains, primarily in energy, through new innovative products based on renewable resources, through agricultural production and sustainable food systems, by enhancing the basic needs of people with the elimination of social inequalities, and by prompting social capital. Other important areas are innovative transformations into low-carbon and circular economy, including waste management, international cooperation, and environmental protection and climate change mitigation. By means of qualitative content analysis, these responses were initially classified into 49 categories followed by the construction of 12 key categories of the most promising success stories depicted in Fig 1. Seeking objectivity in the coding process, the first author of this article and a second coder independently coded the qualitative data. Similar patterns of categorization were reached with a Kappa value of above 80%, adding further weight to the results of this survey. Fig 1 shows that 40% of international experts mentioned success stories related to bio-based energy like biofuel or biogas. Irrespective of ongoing debates about the sustainability of bio-based energy, this finding probably mirrors the substantial efforts that have been made in recent years in many countries [43]. Together with further promotion of solar, wind, and hydropower-based energy, many experts also appreciated the expansion in bioenergy that is primarily based on industrial and agricultural wastes, .e.g. “*next generation biofuels from industrial waste streams”*,*“development of rural bioenergy (biogas) from agri-industrial waste”*, *and “sustainable production of renewable energy from by-products of the sugarcane industry*, *i*.*e*. *from bagasse”*.

30% of respondents also anticipated a great leap forward in bio-based products and materials as upcoming pioneers of the bioeconomy (Fig 1). Sustainable solutions for replacing fossil-made chemicals and other *non-renewable raw materials* with environmentally friendly renewable materials are seen as essential for achieving bioeconomy goals. The shift towards bio-based fibres and materials from both terrestrial and marine sources were emphasized in this respect. Regenerative production systems, biomaterials, and biochemicals were mentioned as the basis for multipurpose uses in the future bioeconomy, as well as for inventing new bio-based products including detergents, papers, clothes and textiles, aromatics, new sweeteners, biodegradable plastics, and innovative construction materials. Cases of innovative products anticipated by the respondents include, *e*.*g*. *“turn wood fibre to silk”*, *“marine-biodegradable packaging that helps fight ocean plastic pollution”*, *and “value creation from macroalgae*, *an underutilized resource”*.

Basic needs of people, mentioned by 21% of experts, covers issues related to a sufficient supply of food and water for human consumption as well as reaching zero hunger in the world. They include sustainable food systems with reduced consumption patterns, regional food production, well-developed production of organic food, alternative protein sources such as insects or in-vitro meat, new algae and seaweed-based food and feed, e.g. *“food and feed from seaweed”* and producing *“meat and milk products without livestock”*. To *“reduce and optimize the use of water for agriculture”*, to decentralise water governance and develop sustainable solutions for managing water resources at the provincial level, as well as reducing and optimising agricultural water use are examples of improving water use efficiency within future bioeconomy, e.g. *“developing sustainable solutions for managing regional water resources”*.

Social inequalities and opportunities were emphasized by 19% of respondents as the most important success stories. These include better health services, improved education and training, promotion of awareness related to the fields of ecology and bioeconomy in educational curricula at the school level, social responsibility, new jobs that integrate social and ecological responsibility as part of decision making, improved working conditions, gender equality, as well as reduced inequality within countries, rural development, and poverty reduction in rural areas. Many experts indicated that minimizing social inequalities and boosting opportunities should be an integral part of any future bioeconomy action plan. This can be done in various ways, e.g. *“biosimilar business with strong infrastructure and human resources”*, *“better education with focus on the importance of nature”*, “*improving quality education starting from basic to higher education by emphasizing the potential of local wisdom and raw materials”*, *“increasing farmers' income by creating more added value to the agriculture product and its by-product”*, *and “poverty reduction in Africa and Asia through regional capacity building on bioeconomic production of food*, *fuel*, *pharmaceuticals*, *etc*.*”*.

Two other important success stories, mentioned by 15% of respondents each, lay in the field of biotechnology and bio-based innovation in industry, as well as in agriculture and fishery. Biotechnology and new techniques like genome editing can foster different sectors of the bioeconomy. Applications include further developments in gene therapy, biopharmaceuticals, and bio-based materials for versatile uses, development of food alternatives, or plant varieties with a special emphasis on improved yield and quality e.g. *“the improvement of indigenous crops through biotechnology”*, *“the development of resistant varieties and molecular farming”* and *“500+ well-characterized molecular chassis for bioproduction (beyond yeast)”*. Alongside biotechnology expansion, advances in industrial bio-based innovation are likely to develop a pioneering small and medium enterprise (SME) culture, e.g. *“strong development of new bio-based industries based on abundant local bio-resources”* and “*bio-based innovations make (…) primary sector more resilient to climate change and*, *consequently*, *a more strategic contributor to global food security while increasing value opportunities for the local economies*”. A small number also explicitly urged for the need to direct biotechnology in line with a sustainable bioeconomy e.g. in *“artificial photosynthesis”*, *“big chunk of public R&D towards nature-based sustainability innovation”*, and adopting bio-tissues and stem cell technology to find proper solutions for lingering diseases e.g. *“regenerative medicine based on stem cells”*. Improvements in agriculture and fishery ranged from new plant varieties to smart features like automated workflows and better production methods (including precision agriculture, conservation, and climate-smart agriculture, reducing agricultural loss, and adding value to food crops). The application of science and technology in agriculture and fishery is seen as important to optimize agricultural productivity and plant traits by means of improved seeds, drought and pest resistance, rapid maturing, etc., e.g. “*adoption of improved crops (including genetically modified) for a range of applications”*, and to revive degraded agricultural lands for crop production *“the rehabilitation of degraded and abandoned agricultural land”*.

With respect to policy, strategy, communication, and cooperation related to future bioeconomy, several topics were mentioned by 15% of respondents. These include elaboration of national bioeconomy strategies and bioeconomy implementation in different sectors e.g. chemical, food, feed, and biotechnology industries or in revised material flows, as well as solving global problems through boosting international cooperation and exchange. Setting up a holistic biomass energy strategy and policy support for renewable energy, as well as policy coherence on biomass uses were also mentioned in this context. Other highlighted topics comprise increased regional support for bioeconomy, establishing platforms for networking and development of public-private partnerships in bioeconomy, together with reinforcing collaboration for better outcomes and efficiency of bio-based industry. In cooperation with developed nations, some developing countries are working to strengthen R&D by allocating funds and resources to reinforce local capabilities and research projects in the sustainable use of biodiversity. This also implies governmental and NGO initiatives to establish a portfolio that fosters and empowers SMEs that produce goods and services derived from biodiversity, e.g. *“better outcomes and efficiency of innovation infrastructures through collaboration*”, *“policy coherence on biomass use”* and *“increased regional support for bioeconomy”*

14% of the experts indicated that the future bioeconomy should consider and promote sustainability, low-carbon and circular economy, together with two related domains: waste management and sidestreams (14%); and environmental protection and climate change (11%) (Fig 1). These interconnected categories cover a wide range of promising success stories of future bioeconomy including the fields of sustainability, reduction of greenhouse gas (GHG) emissions and carbon sequestration, waste management, by-products and sidestream uses, sustainable use of terrestrial and marine resources, and overall responsibility for environmental biodiversity protection and climate change mitigation. An increasing interest in circular economy and related areas of action are on the rise in a growing number of countries. That is principally to ensure coherence and congruence between industrial production, waste management, environmental and climate course, and energy solutions. This ultimately helps to create an optimal environmentally-friendly business model that takes into consideration biodiversity protection, substantial reduction of GHG emissions and their industrial use, reduction of dependency on offshore land, job creation, and innovation. Implementing such a holistic model would therefore represent a major breakthrough to achieving sustainable bioeconomy. In this context, several examples were specified by many respondents, e.g. “*development of bioeconomy clusters comprising activities from R&D to industrial applications; implementation of regional bio-based circular economies”*, *“low-emissions economy*, *reconciling demands for sustainable agriculture and food security”*, *“utilization of agricultural waste and/or by-products and turning them into higher value-added substances using as components in bio- pharmaceuticals*, *food/feed*, *cosmetics or even in medicine”*, *“increase in recycling and conversion of waste to energy”*, *“no waste*, *resource effective production processes and upgrading of all sidestreams”*, *“use of waste gasses including CO2 (and syngas) as feedstocks for biofuels and chemical production*, *with higher yields enabled through co-feeding of green electricity/electrons” and “sustainable use of biodiversity”*.

The technological development in terms of processing and machinery was explicitly mentioned by 5% of respondents. A few innovative solutions were emphasized such as the digitalisation of bioeconomy services and activities and smart irrigation technology, e.g. *“effective machine learning systems for scaling bioproduction”*. Though the idea of a green city delineated as a flagship project candidate in the GBC’s international Delphi study in 2014 [44], only a few experts in the present study (4%) indicated that future bioeconomy should consider the issues of eco-design and green living spaces, such as vertical farming in cities and urban gardening, green metropolitan cities, and reconciling living in large cities with health and bioeconomic principles, e.g. *“new smart and sustainable city environments”*

**Fig 2:**
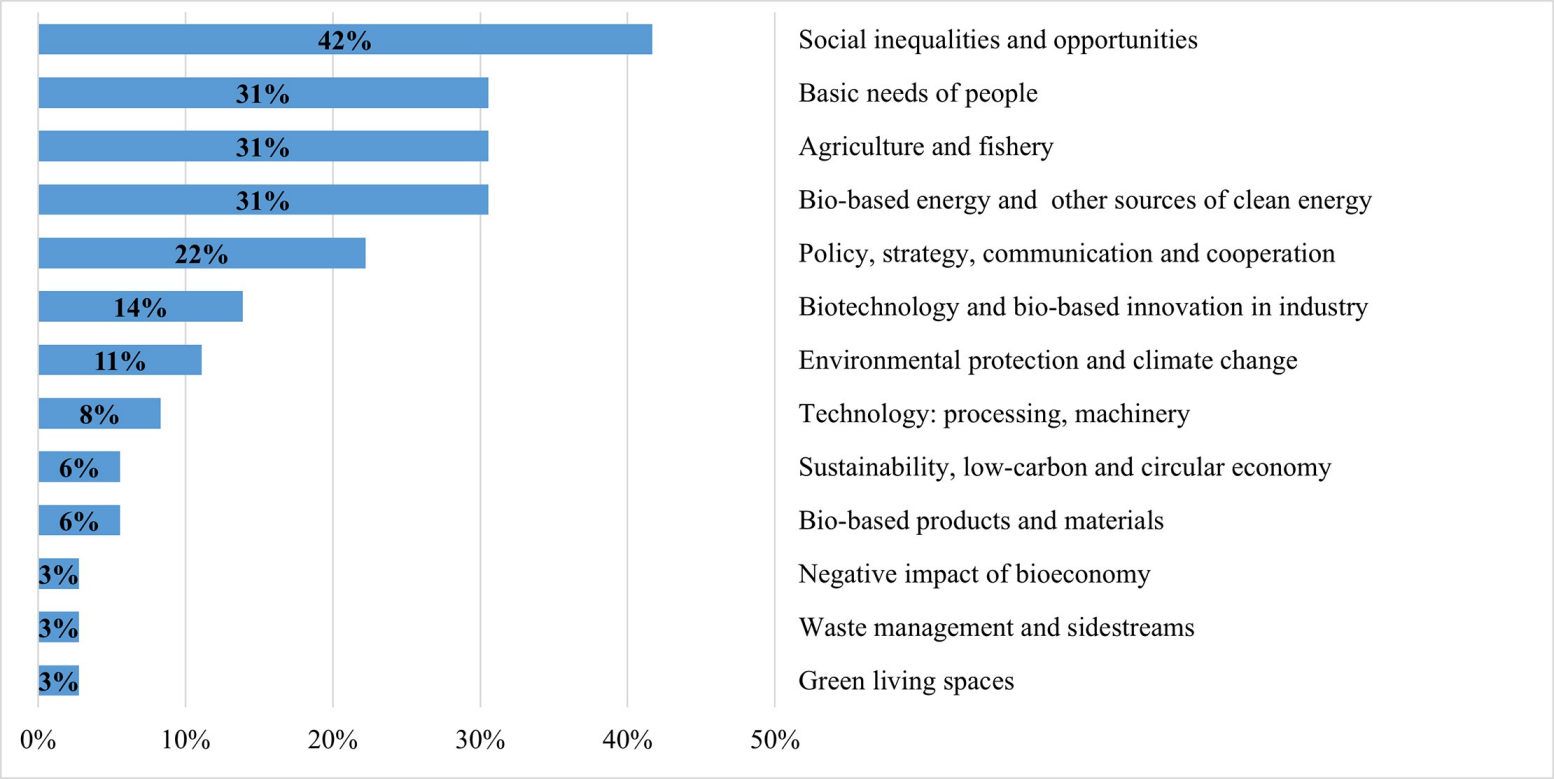
Cluster 1: Main promising success stories of the bioeconomy over the next 20 years in Low- and Lower-Middle-Income Economies (up to $3,955 per capita, n = 36). Note: multiple answers.

**Fig 3:**
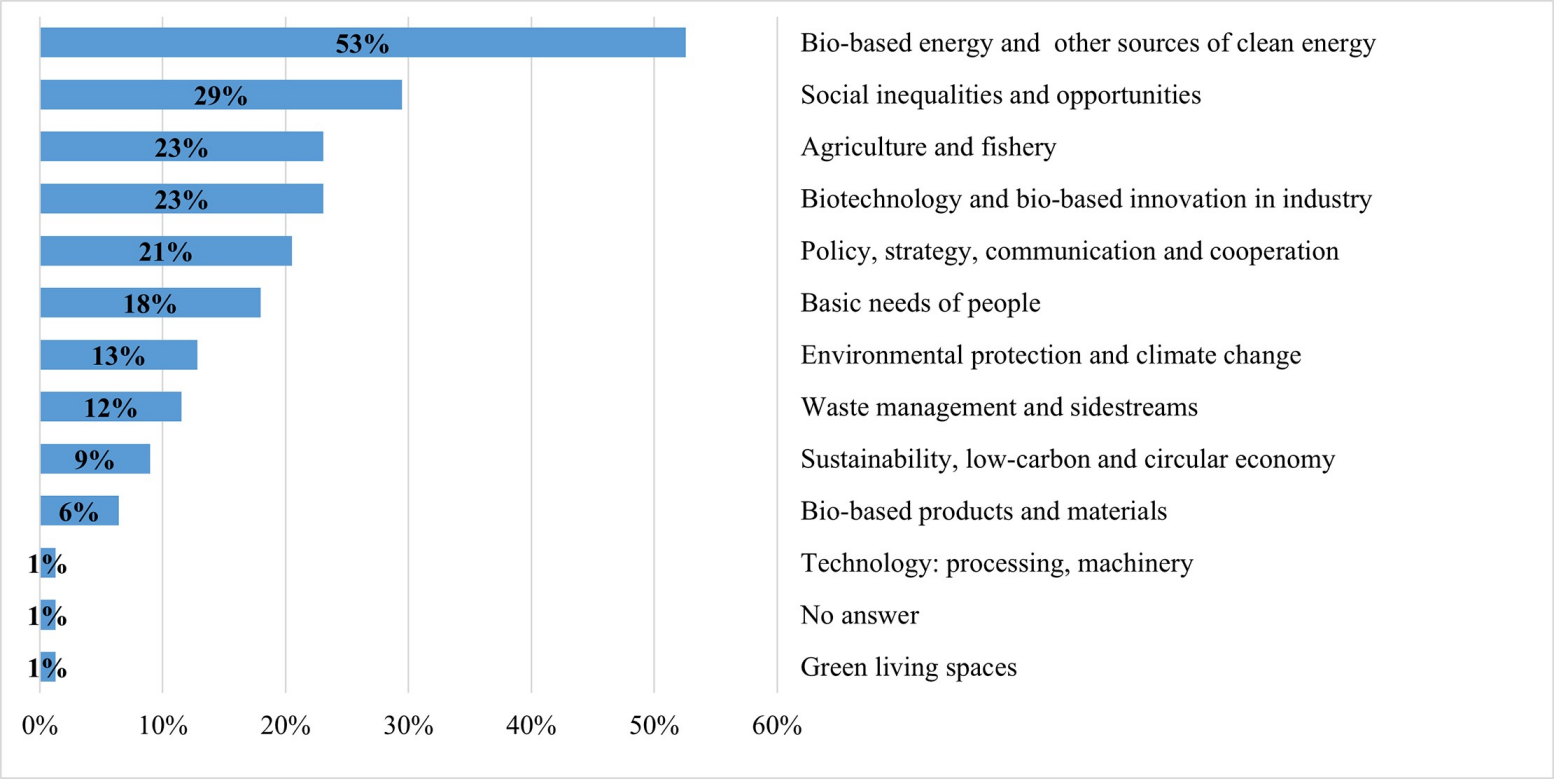
Cluster 2: Main promising success stories of the bioeconomy over the next 20 years in Upper-Middle-Income Economies ($3,956–12,235 per capita, n = 78). Note: multiple answers.

**Fig 4:**
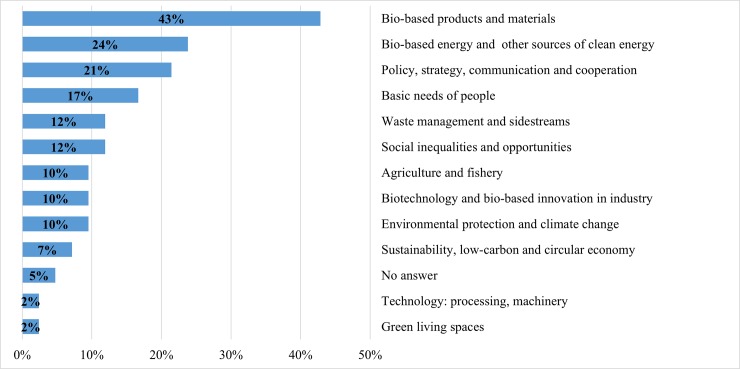
Cluster 3: Main promising success stories of the bioeconomy over the next 20 years in High-Income Economies ($12,236–32,290 per capita, n = 42). Note: multiple answers.

**Fig 5:**
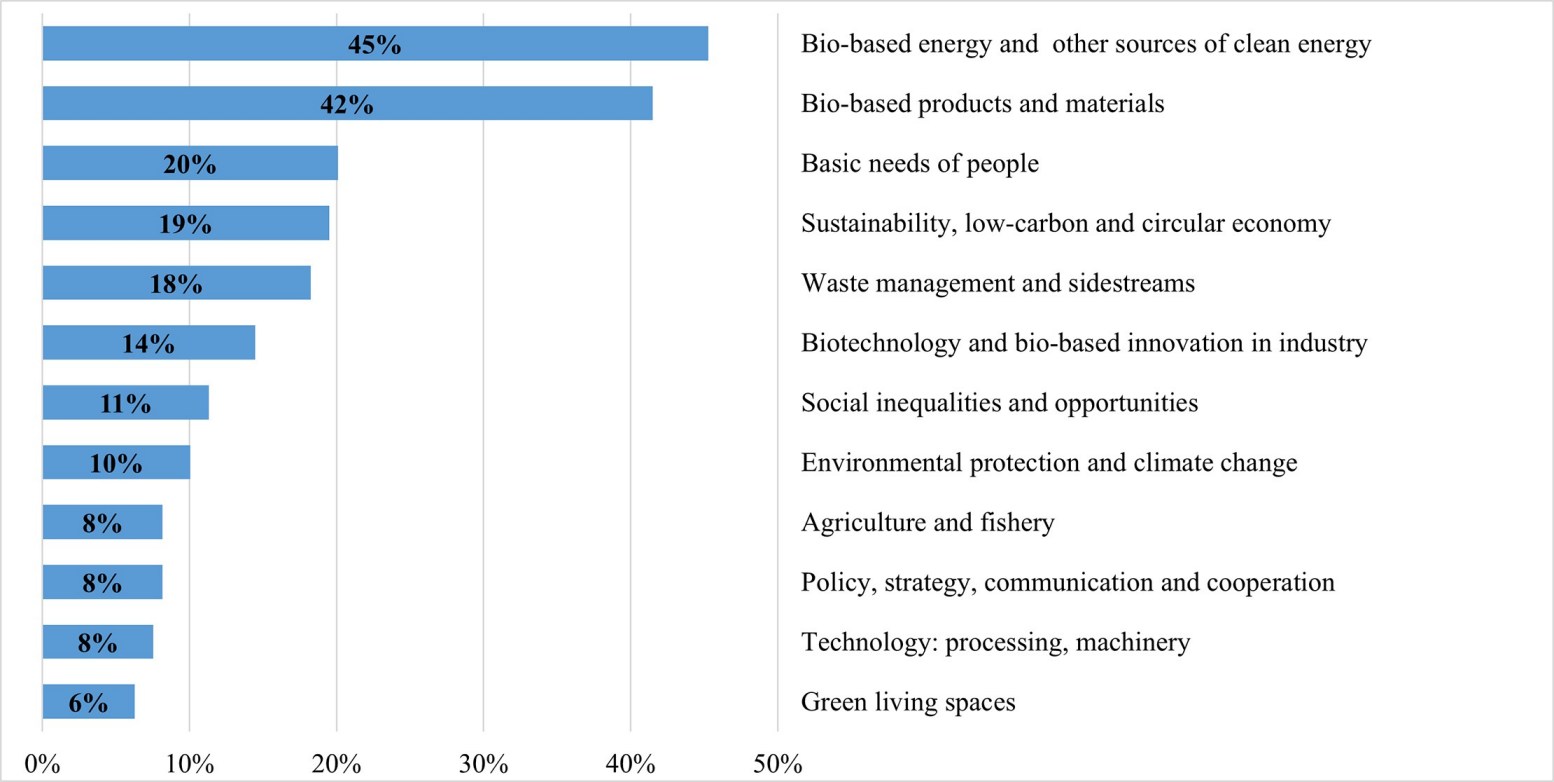
Cluster 4: Main promising success stories of the bioeconomy over the next 20 years in Very-High-Income Economies ($32,291 and more per capita, n = 159). Note: multiple answers.

**Fig 6:**
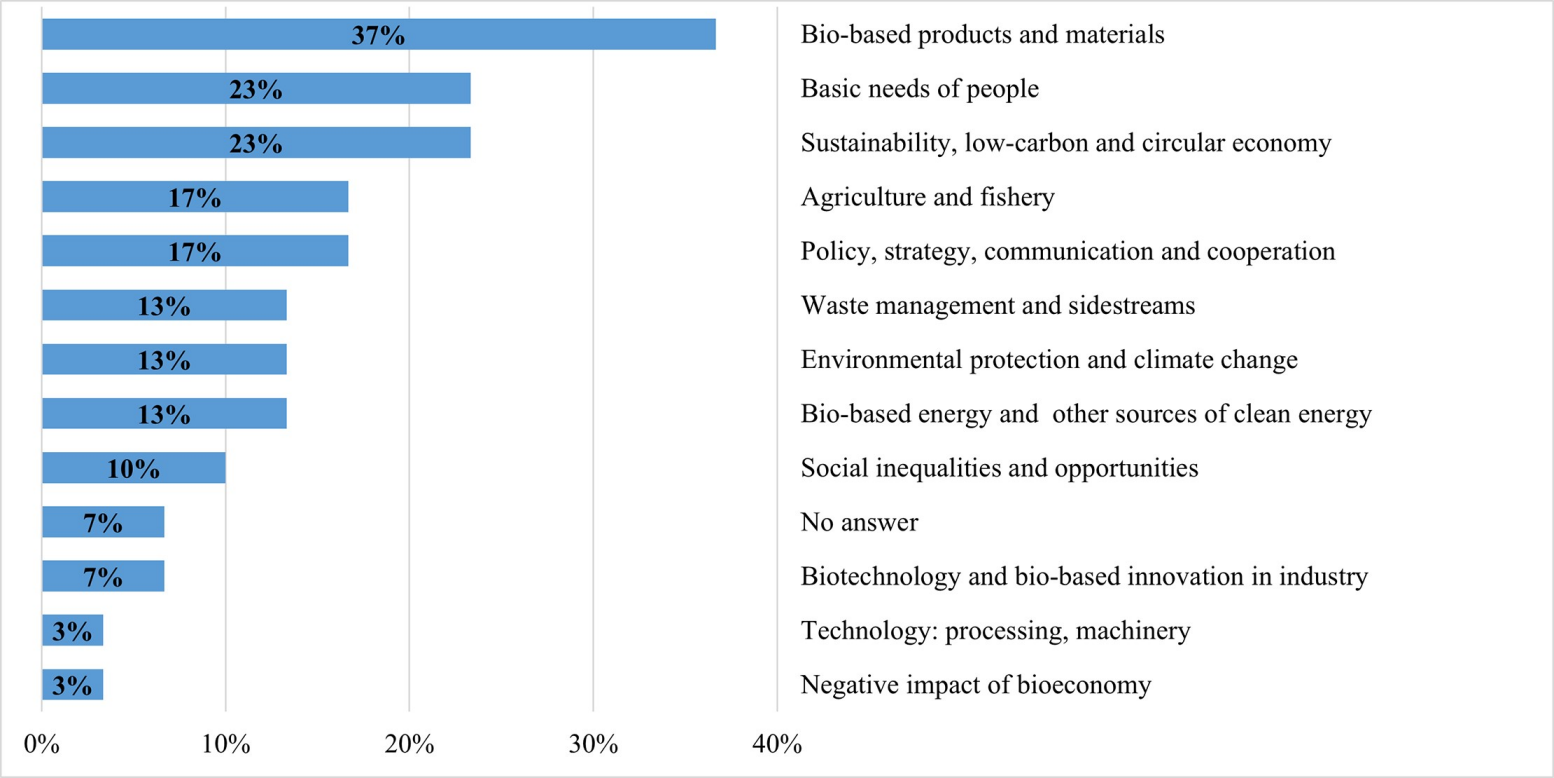
Cluster 5: Main promising success stories of the bioeconomy over the next 20 years in European and International Organisations (n = 30). Note: multiple answers.

Figs 2–6 demonstrate how the proposed clusters differ in terms of the main promising success stories of future bioeconomy. Though the clusters vary substantially in size, major differences amongst the clusters can still be observed, however with different nuances in the priorities that national bioeconomies should undertake over the next 20 years. The following results reveal the most important scenarios of future bioeconomy at the cluster level:

- **Cluster 1: Low- and Lower-Middle-Income Economies (up to $3,955 per capita), (n = 36)**: many experts in this cluster placed greater importance on bioeconomic success stories that eliminate social inequalities and promote social capital (42% of experts), followed by providing for the basic needs of people (e.g. water sanitation, health care, and staple food), improving agricultural and fishery production (31% of each). The importance of these domains come as no surprise since almost all countries in this cluster are still struggling to feed their ever-growing populations [1]. Interestingly, bio-based energy (31%), international cooperation and communication about the field of bioeconomy (22%), as well as biotechnology and bio-based industrial innovations (14%) are also evolving in these countries.
- **Cluster 2: Upper-Middle-Income Economies ($3,956–12,235 per capita), (n = 78)**:

Most experts (52%) appreciated bio-based energy (particularly biofuel) and other sources of clean energy. Like Cluster 1, many experts in this cluster also perceived that future bioeconomy should incorporate the following into its agenda: the elimination of social inequalities and improving social capital (29%), improving agricultural and fishery systems (23%), and to a lesser extent, the basic needs of people (18%). In many countries in this cluster, growing interest can also be foreseen for biotechnology and bio-based innovation in industry (23%), and policy, strategy, communication and cooperation (21%).

- **Cluster 3: High-Income Economies ($12,236–32,290 per capita), (n = 42)**:

A majority of 43% of the experts preferred bio-based products and materials (e.g. biodegradable plastics). About one quarter of the members of Cluster 3 indicated success stories regarding bio-based energy and other sources of clean energy (e.g. a third-generation biorefinery based on agricultural raw materials and vegetable waste), followed by policy, strategy, communication and cooperation (21%) (e.g. publication of national bioeconomy strategy). Basic needs of people (mainly including alternatives for food and feed such as the growth of micro and macro algae for advanced/functional food and feed) came in fourth place with 18% of experts indicating it.

- **Cluster 4: Very-High-Income Economies ($32,291 and more per capita), (n = 159)**:

As the largest cluster of our international sample, cluster 4 has significant impact on the overall result obtained from this survey and on the general conclusions. Most experts in Cluster 4 essentially appreciated the fields of bio-based energy and other sources of clean energy (45%) and bio-based products and materials (43%). Sustainability, low-carbon and circular economy (19%) and waste management and sidestreams (18%), biotechnology and bio-based innovation in industry (14%) also received significant attention from many respondents. Interestingly, 20% of experts envisaged basic needs of people as important and it came in third place (e.g. better quality of food and feed and protein extraction from unused resources) and to a lesser extent, social inequalities and opportunities with 11% of respondents listing it (e.g. social responsibility, gender equality, and better education with focus on the importance of the ecosystem and biodiversity).

- **Cluster 5**: **European and International Organisations (n = 30)**

Many of the officials of the European institutions and international organisations interviewed primarily favoured bio-based products and materials (37%) (e.g. lignin-based carbon fibre, pharmaceuticals from marine sources), followed by securing basic needs of people (23%) (e.g. developing sustainable solutions for managing regional water resources), sustainability, low-carbon and circular economy (23%) together with improvement in waste management (13%) (e.g. promoting circular economy concepts in cities, value-addition to primary agricultural products). Other important success stories included agricultural and fishery systems (17%), and further development in communication and cooperation between countries about sustainable bioeconomy (17%).

### 3.3 Sustainable Development Goals affected strongly

**Fig 7:**
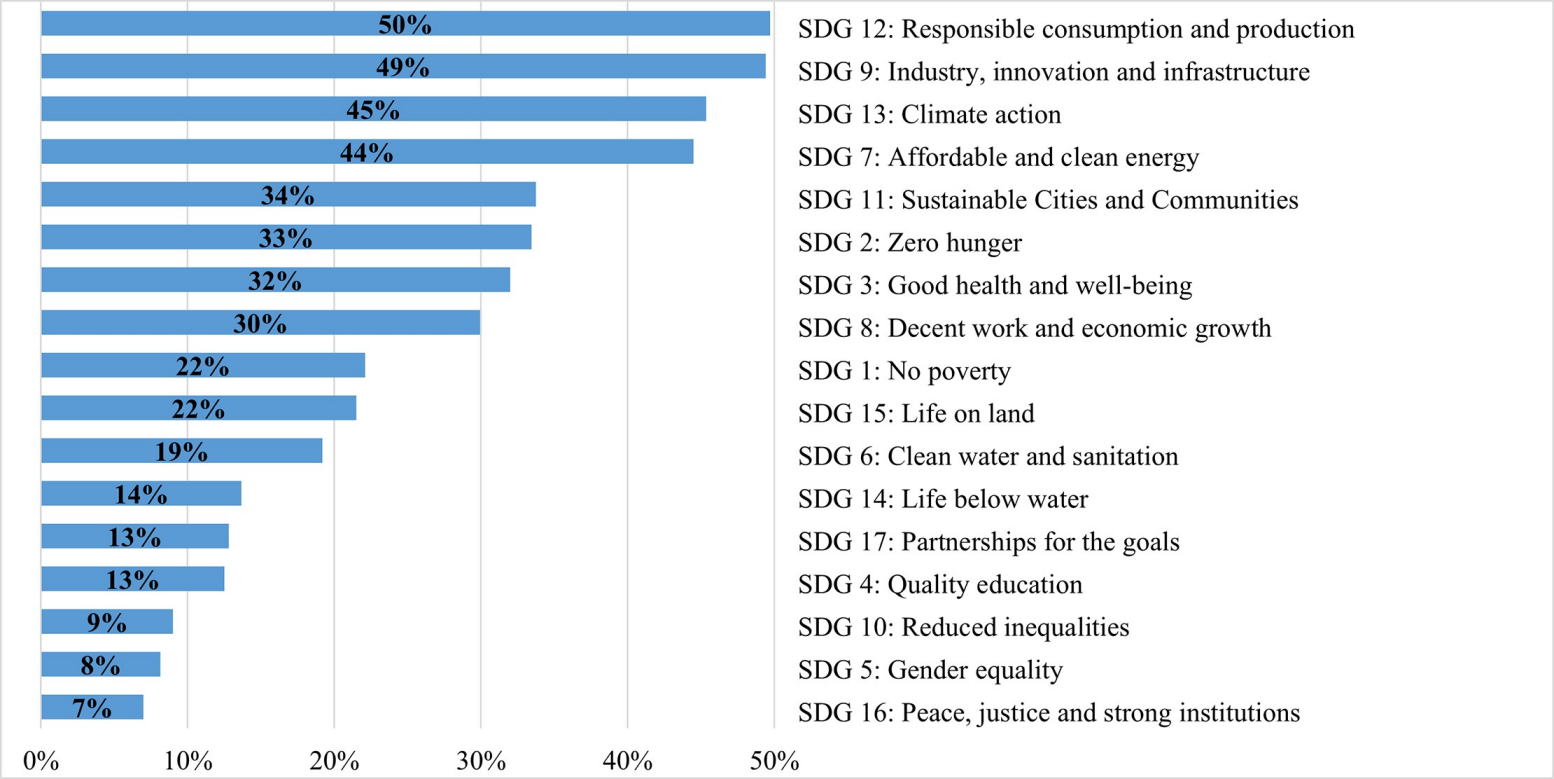
UN Sustainable Development Goals (SDGs) affected (n = 344). Note: multiple answers.

When asked about the UN SDGs that will be affected by promising bioeconomy success stories, experts included every SDG to a certain extent. Fig 7 illustrates the relative importance of the UN SDGs that should be considered in any future bioeconomy approach. Four SDGs stood out within the sample. About 50% of respondents principally indicated that responsible consumption and production (SDG 12) and industry, innovation and infrastructure (SDG 9) should be the main priorities in future bioeconomy, and about 45% of them favoured two other important concerns of humanity, namely climate action (SDG 13) and affordable and clean energy (SDG 7). Other goals such quality education (SDG4), reduced inequalities (SDG 10), gender equality (SDG 5), peace, justice, and strong institutions (SDG16) received the least attention from respondents in the international sample (Fig 7).

Table 3 illustrates how clusters differ regarding the SDGs. Looking at Cluster 1 which includes countries with low income, the three most important SDGs for this cluster are zero hunger, no poverty, and good health and well-being, while experts in Cluster 2 mainly preferred affordable and clean energy, followed by industry, innovation and infrastructure. Like Cluster 1, experts in Cluster 2 were also concerned about eliminating poverty in their countries. Many experts of wealthier countries (Cluster 3 and Cluster 4) primarily appreciated two sustainable development goals related to responsible consumption and production, and industry, innovation and infrastructure. Climate action and producing affordable and clean energy were also main concerns for many experts of both clusters. Unsurprisingly, these results reflect the growing debate in industrialized countries about the issues of sustainable consumption and new food systems, climate change, as well as the role of innovation-based solutions to transform industries into more sustainable and circular economy. Like Clusters 3 and 4, officials of European institutions and international organizations placed special emphasis on responsible consumption and production, climate change mitigation, and promoting industry, innovation and infrastructure. Many of these officials were concerned about finding solutions to achieve zero hunger around the world.

**Table 3:** Importance of Sustainable Development Goals (SDGs) by clusters.

UN SDGs	Cluster 1 (n = 36)	Cluster 2 (n = 78)	Cluster 3 (n = 41)	Cluster 4 (n = 159)	Cluster 5 (n = 30)	Notes
**SDG1: No poverty**	**41,7%**_**a**_	**34,6%**_**a,b**_	**14,6%**_**b,c**_	**12,6%**_**c**_	**26,7%**_**a,b,c**_	**C4 & C5:** **p < .10**
**SDG2: Zero hunger**	**44,4%**_**A**_	**32,1%**_**A,B**_	**19,5%**_**B**_	**32,7%**_**A,B**_	**46,7%**_**A**_	** **
**SDG3: Good health, well being**	44,4%_a_	32,1%_a_	24,4%_a_	32,1%_a_	26,7%_a_	
**SDG4: Quality education**	**22,2%**_**A**_	**20,5%**_**A**_	**7,3%**_**A,B**_	**9,4%**_**B**_	**3,3%**_**B,C**_	
**SDG5: Gender equality**	19,4%_a_	9,0%_a_	4,9%_a_	6,3%_a_	6,7%_a_	
**SDG6: Clean water and sanitation**	22,2%_a_	10,3%_a_	14,6%_a_	23,3%_a_	23,3%_a_	
**SDG7: Affordable and clean energy**	33,3%_a_	46,2%_a_	34,1%_a_	50,9%_a_	33,3%_a_	
**SDG8: Development work**	16,7%_a_	30,8%_a_	29,3%_a_	32,7%_a_	30,0%_a_	
**SDG9: industry, innovation and infrastructure**	**27,8%**_**a**_	**34,6%**_**a**_	**43,9%**_**a,b**_	**63,5%**_**b**_	**46,7%**_**a,b**_	**C3 & C4:** **p < .10**
**SDG10: Reduced inequalities**	5,6%_a_	14,1%_a_	7,3%_a_	6,9%_a_	13,3%_a_	
**SDG11: Sustainable cities and communities**	**13,9%**_**a**_	**21,8%**_**a**_	**22,0%**_**a**_	**47,2%**_**b**_	**33,3%**_**a,b**_	
**SDG12: Responsible consumption and production**	**19,4%**_**a**_	**30,8%**_**a**_	**36,6%**_**a**_	**66,7%**_**b**_	**63,3%**_**b**_	
**SDG13: Climate action**	**36,1%**_**a,b**_	**33,3%**_**a**_	**31,7%**_**a**_	**56,0%**_**b**_	**50,0%**_**a,b**_	
**SDG14: Life below water**	**8,3%**_**a,b**_	**3,8%**_**a**_	**12,2%**_**a,b**_	**19,5%**_**b**_	**16,7%**_**a,b**_	
**SDG15: Life on land**	**11,1%**_**a,b**_	**11,5%**_**a**_	**22,0%**_**a,b**_	**26,4%**_**b**_	**33,3%**_**b,c**_	**C1 & C5:** **p < .10**
**SDG16: Peace, justice and strong institutions**	11,1%_a_	9,0%_a_	4,9%_a_	6,3%_a_	3,3%_a_	
**SDG17: Partnerships for the goals**	13,9%_a_	10,3%_a_	12,2%_a_	13,8%_a_	13,3%_a_	

multiple answers. Significant differences are in bold. Significance level for upper case letters (A, B, C, D): p < 0,1 (they are also italicized) and significance level for lower case letters (a, b, c, d): p < 0,05. Values in the same row and sub-table not sharing the same subscript are significantly different in the two-sided test of equality for column proportions. Tests assume equal variances. Tests are adjusted for all pairwise comparisons within a row of each innermost sub-table using the Benjamini-Hochberg correction.

Table 3 also provides the results of a significance test for the differences among clusters regarding the UN SDGs. Sustainable development goals that showed significant differences (at 2 Alpha levels: 0.05 and 0.10) among the clusters are in bold. Significance differences can basically be observed when comparing countries of low income (Cluster 1) and, to a certain extent, countries of middle income (Cluster 2) with the very-high-income economies (Cluster 4). No poverty (SDG 1), responsible consumption and production (SDG 12), industry, innovation, and infrastructure (SDG 9), and sustainable cities and communities (SDG 11) differ significantly (p< 0.05) between these groups. Significant differences can also be detected between groups (Clusters 3 and 4), while no significant results were found between Clusters 4 and 5 (Table 3).

### 3.4 Public research fund allocation

**Fig 8:**
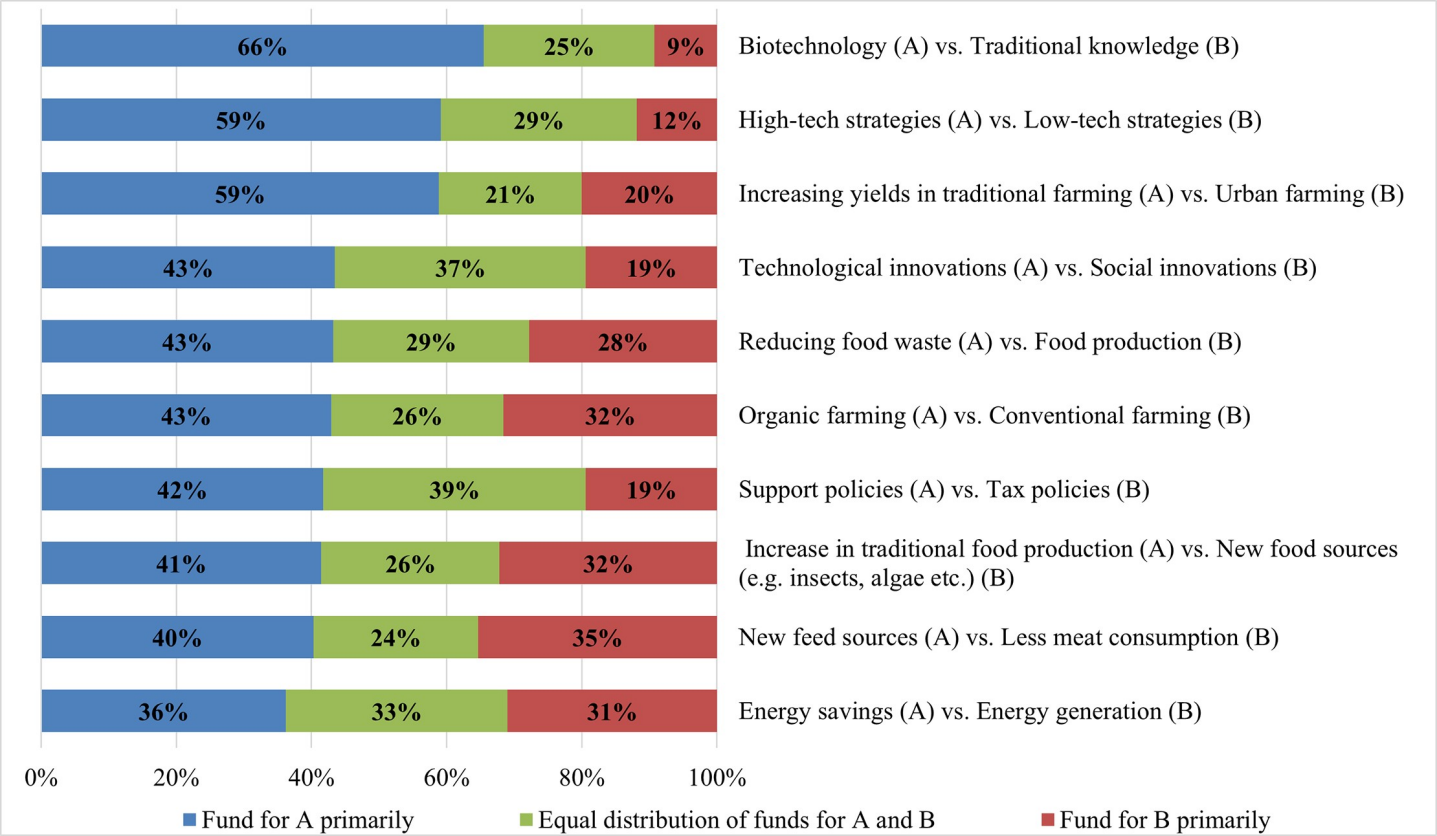
Investment of public research funds in alternatives for future bioeconomy strategies (n = 345).

Upon asking the international experts about the relative importance of a set of 10 pairs of research goals regarding future bioeconomy strategies (Fig 8), technological advances were of prime interest, followed by increasing production and yields in traditional farming. Funds to support policies, increase traditional food production, new feed sources, and to a certain extent, energy savings were also perceived as primarily essential in the overall sample (Fig 8).

Irrespective of clusters and countries, most experts would direct future public research funds mainly towards improvements in the realm of biotechnology, high-tech strategies, and technological-based innovations, rather than towards the domains of traditional knowledge, low-tech strategies, and social innovations, respectively (see Fig 8). Such trends reflect the faith that (bio-) technological advancement can play a major role in developing solutions for a better and more sustainable bioeconomy. Increasing yields in traditional farming (including conventional farming) seems to remain the key pillar of future bioeconomy. Interest in organic farming, however, is high (Fig 8).

Looking at the cluster level, most experts in all clusters favoured yield improvement in traditional farming rather than urban farming. This finding, however, was more pronounced for countries with lower incomes. Low- and middle-income countries still have a major preference for increasing traditional food production and for opening new feed sources rather than decreasing food waste, reducing meat consumption, or looking for new food sources such as insects and algae. This pattern is, however, reversed in the very-high-income economies, who favour new food systems based upon reduction in meat consumption, alternative food sources, and reduced food waste. These views are also shared by the experts of the high-income countries and the European and international organisations, although to a lesser extent. Unsurprisingly, these results reflect the fact that food production and feeding ever-growing populations are of prime interest for many low- and middle-income countries, as people in those countries still need to increase their carbohydrate intake, and a rising middle class envisages meat consumption as part of its success story. On the other hand, new food systems relying on consuming less meat, finding new food alternatives and reducing food waste are high on the agenda of a growing number of countries in the developed world.

Most respondents in the European and international organisations cluster, and to a lesser extent, those of high-income countries, preferred to spend public funds primarily for organic farming. Interestingly, the experts of the very-high-income economies would rather divide such grant money between organic and conventional farming equally. A similar pattern can also be observed in the low- and middle-income clusters.

Remarkably, supporting policies for bioeconomy are preferred over tax policies by many experts in all clusters. More nuances, however, are detected in the low- and middle-income countries. One reason for this observation might be that supporting policies can conceivably reach a larger number of consumers, companies, and producers. While conserving energy is generally preferred over energy production in the overall sample, this picture considerably differs amongst the clusters. Whereas many experts of high and very high-income countries and European and international organisations put a special weight on energy savings, experts from poorer countries place strong emphasis on energy generation. This observation might indicate that many developing countries are still facing major problems in producing enough energy (including the lack of proper energy infrastructure), whereas many industrialized countries are primarily concerned about optimising energy use and looking for fossil-based fuel alternatives.

### 3.5 Potentially conflicting goals

Over the recent past, the continuous debate related to bioeconomy has raised important questions about its sustainability and identified potentially conflicting goals in this respect. About 10–15% of worldwide arable land (1.5 billion hectares) is not used for food production, but rather for bioenergy crops or crops for bio-industrial use. Another one third of global crop land is used to produce feed for meat, milk, and egg production, mainly in industrialized and emerging countries. There is criticism that world-market prices for plant-based food, therefore are increasing, making the situation worse for the poor. Similarly, there is growing criticism that virgin forests, e.g. in South East Asia and Latin America, are being converted into agricultural land to produce e.g. palm oil, soybeans, or beef for export. These three conflicting goals were examined by the experts. Table 4 shows that over three quarters of the respondents in the overall sample admitted the need to address these problems in bioeconomy strategies. Looking at the cluster level, there are only small differences between them. While the wealthiest countries and officials at European and international organisations strongly agreed that non-food uses of arable land, conversion of virgin forests into agricultural land, and use of crop land to produce feed should be addressed in future bioeconomy strategies, the cluster of low-income countries showed relatively less attention to these conflicting bioeconomy goals. Clusters of middle income (Cluster 2) and rich countries (Cluster 3) showed relatively similar patterns of response that lie in the middle between the clusters of the wealthiest and the poorest. An interesting exception can, however, be seen in Cluster 2 regarding conversion of virgin forests into agricultural land, where roughly 90% of experts in this cluster stressed the importance of addressing this conflicting goal in any future bioeconomy path. This result is not surprising, since many countries in this cluster (such as Brazil, Malaysia, and Indonesia) currently suffer strongly from conversion of rainforests into agricultural land for bioenergy purposes.

**Table 4:** Should future bioeconomy strategies deal with the following conflicting goals?

	Cluster 1 (n = 36)	Cluster 2 (n = 78)	Cluster 3 (n = 42)	Cluster 4 (n = 159)	Cluster 5 (n = 30)	Overall sample (n = 345)
**non-food uses of arable land**	66,7%	70,5%	71,4%	80,5%	96,7%	**77,1%**
**use of crop land to produce feed for meat, milk and egg production**	69,4%	73,1%	71,4%	79,9%	83,3%	**76,5%**
**conversion of virgin forests into agricultural land**	75,0%	88,5%	78,6%	86,8%	96,7%	**85,8%**

A wide range of ideas was proposed by the experts. Irrespective of countries and clusters, many experts indicated that future bioeconomy should adopt a holistic approach, improve land use, or follow the food first principle to deal with non-food use of arable land. Among other ideas, valorisation and reduction of waste, looking for alternative resources for bioenergy, and bioindustrial products were also central in this context. Furthermore, most of the experts agreed on the idea that reducing food loss and waste is crucial to eradication of world hunger. Again, the proposed solutions relied greatly on innovation and technological development.

To deal with arable land use for feed, many experts suggested reduction of meat intake, food and feed alternatives, e.g. insects, algae, or an increase in yields and productivity. Setting up policies and regulations, e.g. taxes, incentives, improvement in technology, or regional approaches were also important to resolving this conflicting goal. Lastly, to resolve the conflicting goal related to conversion of virgin forests, many experts emphasized the role of regulation and conservation policies. Sustainability, holistic, and regional approaches were also important in this respect.

## 4 Discussion

Findings of the expert survey indicate that bioeconomy can be seen as vital for realizing many of the UN SDGs. Bio-based innovations are key in this respect. Even though all UN SDGs will be affected by future bioeconomy success stories, four SDGs stood out within the sample: SDG 12: ‘responsible consumption and production’; SDG 9: ‘industry, innovation and infrastructure’; SDG 13: ‘climate action’; and SDG 7: ‘affordable and clean energy’.

Several experts in the present study implicitly or explicitly indicated that the upcoming bioeconomy should consider innovative modifications to the approach in which energy is derived from biomass to escape the food vs. fuel discussion and to circumvent the non-food use of arable land. Ideas include the use of agricultural and woody residues as well as non-woody biomass such as algae, seaweed and switchgrass as substantial bioenergy feedstocks to produce biofuels. Such an option should be favourable to generating a clean fuel that is renewable and environmentally friendly. Therefore, bio-based innovations should consider chemical and biological processes and the application of biotechnology to convert diverse waste streams into third generation biofuels (e.g. lignocellulosic ethanol). Additionally, innovative bioproducts that have versatile applications (e.g. new clothing fibres) should be delivered. These findings are consistent with previous studies in that biofuel from other biomass alternatives can be produced at lower economic and ecological costs in comparison to traditional bioenergy crops such as oil palm and maize (e.g. [45–48]). Similarly, bioeconomy can contribute to overcoming a prominent challenge for growing populations, namely wastewater in urban areas. By means of certain bacterial strains, bio-hydrogen can be produced by co-digestion of domestic wastewater and biodiesel industry effluent [49]. According to experts, further development in other renewable energies resources (solar, wind, and hydropower energies) are also essential for securing sustainable energy resources for future generations. Given technological advances in recent years, solar panels are becoming more efficient and less expensive (e.g. [50–55]). The expansion of solar energy should be further integrated into a holistic energy policy.

Adoption of improved agricultural production and new food systems are another good example towards achieving a sustainable bioeconomy. In this respect, many experts suggested e.g. designing food value chains with no waste, sustainable intensification of food production and sustainable food consumption, new protein alternatives, and balanced diets for all people as well as territorial marketing with organic local agro-food value chains. Other promising success stories lay in bio-based products and materials for production and construction, together with designing green living spaces, e.g. reconciling living in big cities with health and bioeconomic principles as well as growing business ideas in sustainable cities and communities, ecovillages, bio-based constructions and traffic systems in urban areas. It included architectural and building based eco-design, smart cities through use of sensors, and information and communications solutions to enable investment in clean technologies. These findings are in line with the results of a previous international Delphi study conducted by GBC in 2014 [55].

The technical challenges and complexities of a bioeconomic innovation (considering cheaper alternatives under the present economic model) would be a main barrier to realising promising success stories. That is particularly true in the case of bio-based innovations that require maximum technological advances. Nevertheless, many experts perceived various bio-based and recyclable products as having the potential to make the economy more sustainable and lower its reliance on fossil fuels. In contrast to the conventional business model (economic growth, take-make-use-dispose economy, business-as-usual), bio-based circular economy is designed to close the loop and minimize the net extraction of key resources and their accompanied negative environmental impacts on ecosystems [12]. That includes less resource consumption, environmental-friendly design, and easy-to-recycle products. Accordingly, bio-based circular economy pays particular attention to material flow analysis, eco-design, industrial ecology, reverse logistics, and functional economy. Thus, bio-based circular economy perceives old products not as waste, but rather as raw materials for a new product. Political agendas towards bio-based circular economy are on a rise (e.g. [12]). However, more initiatives and steps should be made to boost the concept of bio-based circular economy in all economic sectors. Government supported funding programmes for businesses are needed to help bio-based circular economy to achieve greater environmental performance. Ultimately, bio-based circular economy will signify a great breakthrough to transitioning into a sustainable bioeconomic model that will be more robust and resilient for future generations.

Though the road to sustainable bioeconomy is still long, innovative solutions for existing problems can help to shift attention in the right direction towards a better world. Many promising bio-based innovations are already present, and future (bio)technological advances can facilitate the realization of many of them on a commercial scale. An example discussed worldwide are biodegradable plastics, suitable for a multitude of uses, including manufacturing environmentally friendly packaging. In contrast to fossil-based plastics, bioplastics are degradable and have a significantly lower carbon footprint.

Expanding bioeconomy research and directing more funds towards innovation endorsement could be useful tools for many countries to advance the management of its renewable biological resources in an efficient and sustainable manner. It can also help to open new and diversified markets for food and bio-based products. Without agreed-upon global outlines and priorities, it is not possible to achieve a sustainable bioeconomy sector at a global level. Joint action and collaboration among nations (at both governmental and NGO levels) are therefore required to improve the global investments in information collection and assessment methods regarding the national bioeconomy. Advanced countries in bioeconomy can play a vital role and make an important contribution at a global level. Knowledge sharing and information exchange among nations will be important to spreading sustainable bioeconomic innovations and bioeconomy-related strategies, particularly in developing and less developed countries.

## 5 Conclusions

Overall, survey results suggest that more efforts and initiatives should be undertaken to raise the awareness of, and thus, later to convince policy-makers and people (of different nations) of the viability of such success stories for a more sustainable circular bioeconomy. Accordingly, a prototype shift in economy and society (primarily abandoning “business-as-usual” together with consumer behaviour change) is needed to achieve a sustainable bioeconomy and the related UN SDGs. This requires a new paradigm for global bioeconomy that emphasizes the powers of ecosystems, in which everything cascades and nothing is made for only one purpose, thus resulting in a “no waste” business model. Zero waste and zero emissions are ambitious goals for a more sustainable bioeconomy. Therefore, further initiatives and innovations to reduce waste and emissions should be encouraged. Redesigning the extant economic model (at both the production and consumption levels) into industrial clusters inspired by natural systems is important for a sustainable global bioeconomy.

Tangible measures of sustainable bioeconomy need to be developed and its contributions for meeting the UN sustainable development goals must to be tracked and documented at different levels, starting from the local, to the regional, up to the international level. Such an evaluation process needs to be ratified internationally and managed regionally by international organisations (e.g. the UN and its regional subsidiaries). The elimination of fossil-fuel subsidies and the adoption of a global taxing strategy for carbon footprint and other negative impacts on the environment and climate change should be an integral part of any bioeconomy evaluation process, since they are of prime interest to achieving many of the UN sustainable development goals. Respondents from low- and middle-income countries primarily mentioned bioeconomy success stories related to basic human needs and securing affordable energy to meet the primary needs of growing populations. Therefore, elimination of poverty and reaching zero hunger, rural development, and promoting quality education need to be priority targets for future bioeconomy in these countries. Experts from high income countries foresee successes related to renewable energy and bio-based products, responsible production and consumption (new food systems with less meat consumption, less food waste, and new food sources), bio-based innovations, climate change mitigation, as well as circular and low-carbon economy. These trends should be further enhanced and integrated into future bioeconomy strategies.

High income economies should increasingly abandon fossil-based industries by adopting paradigms that are fully based on renewable resources as the mainstay of such transformation processes. GHG emissions can be significantly reduced by implementing an effective carbon strategy that makes carbon polluters pay for the associated environmental problems (including biodiversity loss, ecosystem failure, global warming, and climate change), while offering incentives for those industries adopting business models based on innovative renewable products and energy policies). Sustainable green city concepts and low-carbon circular economy should be further promoted in any future global bioeconomy.

Finally, follow-up studies could help to determine the state-of-the-art regarding the applicability of the proposed success stories of future bioeconomy and the role of bioeconomy in achieving the stated UN SDGs under different contexts around the world. Further studies can also provide ideas for new bio-based innovations better suited under given conditions. Furthermore, as the global bioeconomy summit 2015 inaugurated the debate on bioeconomy policy, which was advanced in the second summit 2018 by opening doors for further international exchange and consultation on the bioeconomy, follow-up summits (at both regional and international levels) would be indispensable platforms for developing further international strategies, together with scientific and political support programmes.
